# Poster Session I - A110 TRENDS IN SPENDING ON ADVANCED IBD THERAPIES PRESCRIBED BY GASTROENTEROLOGISTS IN MEDICARE

**DOI:** 10.1093/jcag/gwaf042.110

**Published:** 2026-02-13

**Authors:** M Raber, C Lu, C Ma, Y Nasser, S Congly

**Affiliations:** University of Calgary Cumming School of Medicine, Calgary, AB, Canada; University of Calgary Cumming School of Medicine, Calgary, AB, Canada; University of Calgary Cumming School of Medicine, Calgary, AB, Canada; University of Calgary Cumming School of Medicine, Calgary, AB, Canada; University of Calgary Cumming School of Medicine, Calgary, AB, Canada

## Abstract

**Background:**

Inflammatory bowel disease (IBD), including ulcerative colitis and Crohn’s disease, affects millions of people worldwide. Advanced therapies including biologics and Janus Kinase (JAK) inhibitors have demonstrated effectiveness in the treatment of IBD, leading to improved quality of life from increased utilization. However, these advanced therapies are associated with a significant economic burden due to their high costs.

**Aims:**

To better understand the economic impact of advanced agents for IBD, we studied gastroenterology prescribing trends of these agents and their associated costs in Medicare.

**Methods:**

Data gathered from Medicare Parts B (covering injections/infusions given in an office setting) and D (covering outpatient medications taken at home) from a 10-year period between 2014 to 2023 to examine the utilization and costs of advanced agents prescribed by gastroenterologists to treat IBD. Prescription volumes, trends, and spending patterns over time for each approved IBD advanced therapy, with a subgroup for patients >65 for the Part D analysis. Potential savings using biosimilars as compared to biologics were estimated. Costs were adjusted for inflation to 2023 USD.

**Results:**

Costs and claims for GI prescribed advanced therapies increased exponentially during the 10-year study period, with total spending increasing from $237.5 million in 2014 to $2.12 billion in 2023. Similar trends in costs and claims for Part D were seen in the > 65 subgroup. Adalimumab initially dominated the market share; ustekinumab after its introduction became the dominant drug and accounted for > 60% of Part D spending in 2023. If biologics were priced at the lowest cost biosimilar, there could be $453 million in savings.

**Conclusions:**

Gastroenterology based utilization and spending on advanced therapies are increasing exponentially. Understanding prescribing habits may help with the development of cost-effective policies.

**Funding Agencies:**

None

